# Induction chemotherapy with CPX-351 in acute myeloid leukemia: revisiting the role of early bone marrow assessment

**DOI:** 10.1038/s41375-025-02675-7

**Published:** 2025-07-03

**Authors:** Julian Ronnacker, Leo Ruhnke, Christoph Röllig, Jan Moritz Middeke, Regina Herbst, Anke Morgner, Julia M. Unglaub, Tim Sauer, Karin Huber, David Baden, Lars Fransecky, Melanie Nogueira Gezer, Martina Crysandt, Edgar Jost, Madlen Jentzsch, Klaus H. Metzeler, Andrew F. Berdel, Marc-André Urbahn, Lina Kolloch, Matthias Stelljes, Uwe Platzbecker, Tim H. Brümmendorf, Claudia Baldus, Carsten Müller-Tidow, Mathias Hänel, Martin Bornhäuser, Klaus Wethmar, Utz Krug, Georg Lenz, Jan-Henrik Mikesch, Christoph Schliemann

**Affiliations:** 1 Department of Medicine A, University Hospital Münster, Münster, Germany; 2 Department of Medicine I, University Hospital TU Dresden, Dresden, Germany; 3 Department of Medicine III, Klinikum Chemnitz, Chemnitz, Germany; 4 Department of Medicine V, University Hospital Heidelberg, Heidelberg, Germany; 5 Department of Medicine II, University Hospital Schleswig-Holstein, Campus Kiel, Kiel, Germany; 6 Department of Hematology, Oncology, Hemostaseology and Stem Cell Transplantation, Medical Faculty, Centre for Integrated Oncology Aachen Bonn Cologne Duesseldorf, Aachen, Germany; 7 Department of Hematology and Cellular Therapy, University Hospital Leipzig, Leipzig, Germany; 8 DKMS Collection Center gGmbH, Cologne, Germany

**Keywords:** Acute myeloid leukaemia, Clinical trials, Leukaemia

## To the Editor:

The liposomal dual-drug formulation of daunorubicin and cytarabine, CPX-351, has become the preferred induction therapy for most patients with therapy-related acute myeloid leukemia (t-AML, now termed AML post-cytotoxic therapy, AML-pCT, per WHO 2022 definitions [1]) and AML with myelodysplasia-related changes (AML-MRC or now AML-MR) who are eligible for intensive chemotherapy. Following evidence of improved survival compared to induction with conventional daunorubicin and cytarabine [2], CPX-351 was approved by the U.S. Food and Drug Administration and the European Medicines Agency in 2018 and 2019, respectively, for patients with t-AML and AML-MRC, and has since shown efficacy in real-world settings [3–6].

For over 25 years, early response assessment by bone marrow (BM) aspiration around day 14 after conventional intensive induction has been routine in many centers worldwide [7–12]. Patients who fail to achieve blast-free aplasia at this time point are more likely to have refractory disease and are at a higher risk of early death [7, 8, 11]. For these patients, salvage chemotherapy aiming to induce remission and to enable subsequent allogeneic hematopoietic cell transplantation (HCT) is often considered standard of care [13]. In many centers, refractory disease at day 14 guides the evaluation and timing of early HCT.

Given the distinct pharmacokinetics of CPX-351, and its use in patients with secondary-type AML, the utility of day 14 BM assessment with CPX-351 induction remains unclear [10]. To address this, we conducted a retrospective multicenter study to investigate whether the assessment of residual leukemic blast count in an early BM assessment around day 14 after initiation of CPX-351 reliably predicts treatment failure. This was facilitated by the common practice in many German centers of administering only a single induction cycle of CPX-351.

Patient data were retrieved from seven centers of the German Study Alliance Leukemia (SAL) following approval by the Ethics Committee of Westphalia-Lippe (AZ 2023-032-f-S) and in accordance with relevant ethical and regulatory guidelines, including the Declaration of Helsinki. Early response was assessed between days 13 and 17 following initiation of CPX-351 treatment (with CPX-351 initiated on day 0) by cytologic evaluation of BM aspirates. Blast counts <5% were defined as early blast clearance. All patients included in the final analysis underwent a definitive remission assessment after one cycle of CPX-351, either upon hematologic recovery or, in refractory cases, no earlier than day 22. Induction failure (IF) was defined as persistent AML (≥5% blasts on definitive BM cytology) after one course of CPX-351, persistence or reappearance of blasts in peripheral blood (except for transient, low-level peripheral blasts during hematological regeneration), or early death prior to final response evaluation. Time-to-event outcomes were analyzed from the day of early or definitive response assessment, as indicated. Multivariable Cox regression and logistic regression models were used to assess the prognostic impact of early blast clearance. Receiver operating characteristics (ROC) curve analysis was used to evaluate the predictive performance and to determine optimal blast count cut-offs, with area under the curve (AUC) comparisons made using DeLong’s test (*pROC* package in R).

Of 173 patients undergoing early BM assessment, 138 were included in the final analysis after excluding those who proceeded to next treatment without confirmatory assessments or with missing data (Supplementary Fig. S1). Early response was evaluated at a median (range) of 14 (13–17) days following the first dose of CPX-351. Median (range) blast count at this time was <5% (0–90%). A total of 100 out of 138 patients (72%) achieved early blast clearance, 20 (14%) patients had a partial response at early response assessment, and 18 (13%) patients showed no response. Baseline and disease characteristics were balanced between groups with and without early blast clearance (Table 1). Definitive remission assessment after one cycle of CPX-351 was performed at a median (range) of 35 (22–75) days after initiation of CPX-351, and 21 (7–58) post early BM assessment. Of the 138 patients, 87 (63%) patients achieved CR/CRi (CR 44%, CRi 19%), three (2%) patients achieved MLFS, while 43 (31%) had refractory disease, and five (4%) died before a definitive response evaluation.

**Table 1:** Baseline and treatment characteristics by early blast clearance.

	All patients (*n* = 138)	Early blast clearance (*n* = 100)	No early blast clearance (*n* = 38)	*P*
Baseline characteristics				
Age, years, median (range)	64 (22–80)	64 (22–80)	63 (38–80)	0.79^a^
Male sex, n (%)	62 (45)	45 (45)	17 (45)	0.98^b^
AML ontogeny, n (%)				0.65^b^
AML-MRC	107 (78)	76 (76)	31 (82)	
t-AML	31 (22)	24 (24)	7 (18)	
ELN 2022 genetic risk, n (%)				0.42^c^
Favorable	6 (4)	5 (5)	1 (3)	
Intermediate	28 (20)	23 (23)	5 (13)	
Adverse	104 (75)	72 (72)	32 (84)	
BM blast count, median (range)	35 (10–91)	35 (13–91)	38 (10–90)	0.74^a^
PB blast count, median (range)	5 (0–96)	5 (0–96)	8 (0–88)	0.14^a^
WBC, x 10^3^/µL, median (range)	3.7 (0–195)	3.5 (0–195)	4.5 (0.7–146)	0.98^a^
Further treatments				
Second CPX-351 induction therapy, n (%)	8 (6)	7 (7)	1 (3)	0.44^c^
Salvage therapy^d^, n (%)	9 (7)	2 (2)	7 (18)	**0.001**^b^
Received HCT, n (%)	110 (80)	84 (84)	26 (68)	**0.04**^b^

*AML* acute myeloid leukemia, *AML-MRC* AML with myelodysplasia-related changes, *BM* bone marrow. *ELN* European LeukemiaNet, *HCT* allogeneic hematopoietic cell transplantation, *PB* peripheral blood, *t-AML* therapy-related AML, *WBC* white blood cell.

^a^Mann–Whitney U test.

^b^chi-squared test.

^c^Fisher’s exact test.

^d^Includes conventional high-dose cytarabine-based salvage therapy (*n* = 3) and non-intensive treatments.

**Fig. 1:**
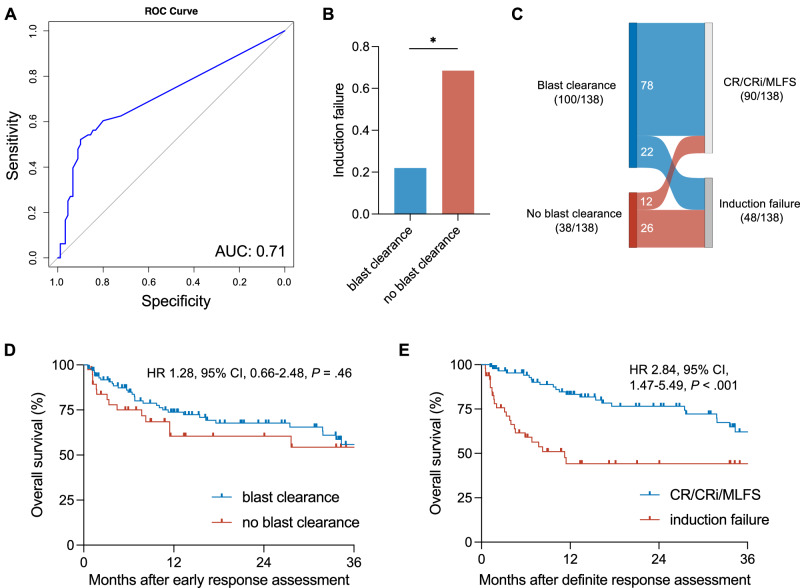
Cut-off definition and outcome by early bone marrow response in patients receiving CPX-351 induction. The predictive value of early response assessment was evaluated by receiver operating characteristics, calculating the AUC and the Youden index. A cut-off of 5% BM blasts was identified as the optimal threshold for predicting induction response (**A**). Patients who failed to achieve early blast clearance (BM blast count <5%) were at higher risk of IF (**B**). Sankey diagram visualizing patient trajectories between early and definitive response assessment (**C**). OS did not differ between patients with and without early blast clearance (**D**) but differed significantly when stratified by definitive response after one course of CPX-351 (**E**). AUC area under the curve, CI confidence interval, CR complete remission, CRi CR with incomplete hematologic recovery, HR hazard ratio, MLFS morphologic leukemia-free state, ROC receiver operating characteristics.

We used ROC analysis to calculate the most decisive cut-off for predicting response to CPX-351 induction by early response assessment (Fig. 1A). The cut-off value for the integer number of BM blasts that maximized the Youden index was 5%, with an AUC of 0.71. This threshold was used to define early blast clearance for further analyses.

Patients who failed to achieve early blast clearance (<5%) were more likely to experience IF (26/38 [68%] vs. 22/100 [22%], *P* < 0.001, Fig. 1B). Notably, twelve of 38 patients (32%) who failed to achieve early blast clearance later achieved CR (*n* = 9) or CRi (*n* = 3; Fig. 1C). The sensitivity and specificity for IF prediction were 26/48 (54%) and 78/90 (87%), respectively, with a positive predictive value (PPV) of 26/38 (68%) and negative predictive value (NPV) of 78/100 (78%) (Supplementary Table 1). These findings remained stable in sensitivity analysis excluding early deaths (Supplementary Table 2). Adopting an alternative threshold of 15% BM blasts to define early blast clearance resulted in an improvement in the sensitivity for identifying patients with IF (73% vs. 54%); however, this was associated with a decline in the negative predictive value for remission (NPV 71% vs. 78%; Supplementary Table 3).

The median (range) follow-up of surviving patients was 24 (17–34) months. Two-year overall survival (OS) was 66% (95% CI, 57–75%). OS did not significantly differ between patients with early blast clearance and those without (hazard ratio [HR] 1.28, 95% confidence interval [CI], 0.66–2.48, *P* = 0.46, Fig. 1D). Similarly, no association of early blast clearance with OS was observed after adjustment for age, ELN 2022 genetic risk, and AML ontogeny in a multivariable analysis (Supplementary Table 4). These results were further substantiated by analysis of the day 14 BM blast count treated as a continuous variable (Supplementary Table 5). However, failure to achieve early blast clearance was associated with significantly increased odds of IF (adjusted odds ratio 8.06, 95% CI, 3.50–19.67, *P* < 0.001). Although limited to a small number of patients, OS in patients with response to induction therapy did not differ significantly whether they had achieved early blast clearance (*n* = 78) or not (*n* = 12; HR 1.75, 95% CI, 0.55–5.58, *P* = 0.44). In contrast, patients with IF at confirmatory remission assessment had significantly reduced OS (HR 2.84, 95% CI, 1.47–5.49, *P* < 0.001, Fig. 1E).

In an exploratory subgroup analysis, we compared early assessments on days 15–17 (*n* = 56) versus days 13–14 (*n* = 82). ROC analysis yielded a higher AUC for assessments on days 15–17 (0.80 vs. 0.61, *P* = 0.040; Supplementary Figs. S2A, B), with improved sensitivity (71% vs. 38%, *P* = 0.02) and comparable specificity (84% vs. 88%, *P* = 0.63; Supplementary Tables 6, 7).

Next, we hypothesized that the reduction of leukemic burden following CPX-351, rather than the absolute blast count at early response assessment, may better predict treatment response and outcome. To test this, we assessed partial response (PR), defined as a >50% reduction in BM blasts around day 14 without achieving blast clearance <5%, following the criteria used in the pivotal study by Lancet et al. [2]. Among 20 patients meeting the definition of PR, 13 (65%) ultimately experienced IF, and OS did not differ from patients who showed no early treatment response (Supplementary Fig. S3).

Early BM assessment is attractive because it is easy to perform and may allow physicians to adjust their treatment strategy early in the event of a poor response to the first induction cycle [9]. While day 14 BM evaluation during conventional AML induction therapy should be considered according to guidelines [14] and is common practice in many centers worldwide, it is important to note that early blast persistence, often used to guide treatment intensification, has low specificity for predicting IF [9, 10]. Prior studies have shown a consistent association between early blast clearance and hematologic remission following conventional induction therapy [7, 8, 15], but its link to survival is less clear. Some large multicenter studies support the association with survival [7, 8], whereas others do not [15, 16]. Therapeutic decisions influenced by early BM results may have confounded survival outcomes. A recent meta-analysis that pooled several publications found a specificity of 92% for <5% blasts at day 14 BM to predict remission after conventional chemotherapy, while the sensitivity to detect patients with IF for ≥5% blasts was 54% [10].

One limitation of our study is the inability to assess marrow cellularity due to the lack of concurrent biopsies, which may have resulted in an overestimation of disease burden in patients with hypoplastic marrows. Previously, it was shown that marrow hypoplasticity is one of the strongest predictor of remission following double induction chemotherapy [12]. However, sensitivity analyses using higher blast cut-offs confirmed the robustness of our conclusions. Additionally, no centralized MRD monitoring was performed, and thus we cannot exclude the possibility that differences in remission depth between CR/CRi patients with or without blast clearance may have contributed to the lack of a significant difference in OS.

Within these constrains, our study demonstrates that early BM assessment following CPX-351 induction provides information comparable to that observed with conventional chemotherapy, despite CPX-351’s distinct pharmacokinetics. Although early blast clearance correlates with remission, its limited sensitivity for predicting IF cautions against premature treatment modifications based solely on early blast persistence. Delaying BM assessment beyond day 14 may improve risk stratification and support more informed clinical decision-making.

## Supplementary information

Supplemental material

## Data Availability

The data that support the findings of this study are available from the corresponding author upon reasonable request.
