# Supplementary material for: Induction chemotherapy with CPX-351 in acute myeloid leukemia: revisiting the role of early bone marrow assessment
Source: Leukemia. 2025 Jul 3;39(9):2266–9. doi: 10.1038/s41375-025-02675-7 (PMC12380596; doi:10.1038/s41375-025-02675-7)
Supplement: Supplementary file 1 — Supplemental material [file 41375_2025_2675_MOESM1_ESM.pdf]

# Supplementary data

## Induction Chemotherapy with CPX-351 in Acute Myeloid Leukemia: Revisiting the Role of Early Bone Marrow Assessment

Julian Ronnacker[1], Leo Ruhnke[2], Christoph Röllig[2], Jan Moritz Middeke[2], Regina Herbst[3], Anke Morgner[3], Julia M. Unglaub[4], Tim Sauer[4], Karin Huber[5], David Baden[5], Lars Fransecky[5], Melanie Nogueira Gezer[6], Martina Crysandt[6], Edgar Jost[6], Madlen Jentzsch[7], Klaus H. Metzeler[7], Andrew F. Berdel[1], Marc-André Urbahn[1], Lina Kolloch[1], Matthias Stelljes[1], Uwe Platzbecker[7], Tim H. Brümmendorf[6], Claudia Baldus[5], Carsten Müller-Tidow[4], Mathias Hänel[3], Martin Bornhäuser[2], Klaus Wethmar[1], Utz Krug[8], Georg Lenz[1], Jan-Henrik Mikesch[1], Christoph Schliemann[1]

[1]Department of Medicine A, University Hospital Münster, Münster, Germany

[2]Department of Medicine I, University Hospital Dresden, Dresden, Germany

[3]Department of Medicine III, Klinikum Chemnitz, Chemnitz, Germany

[4]Department of Medicine V, University Hospital Heidelberg, Heidelberg, Germany

[5]Department of Medicine II, University Hospital Kiel, Kiel, Germany

[6]Department of Medicine IV, University Hospital Aachen, Aachen, Germany

[7]Department of Hematology and Cellular Therapy, University Hospital Leipzig, Leipzig, Germany

[8]Department of Medicine III, Klinikum Leverkusen, Leverkusen, Germany

**Correspondence:** Christoph Schliemann, MD, Head of Leukemia Program, Department of Medicine A, University Hospital Münster, Albert-Schweitzer-Campus 1, 48149 Münster, Germany, Phone +49 251 83 45363, Fax +49 251 83 47588, email christoph.schliemann@ukmuenster.de

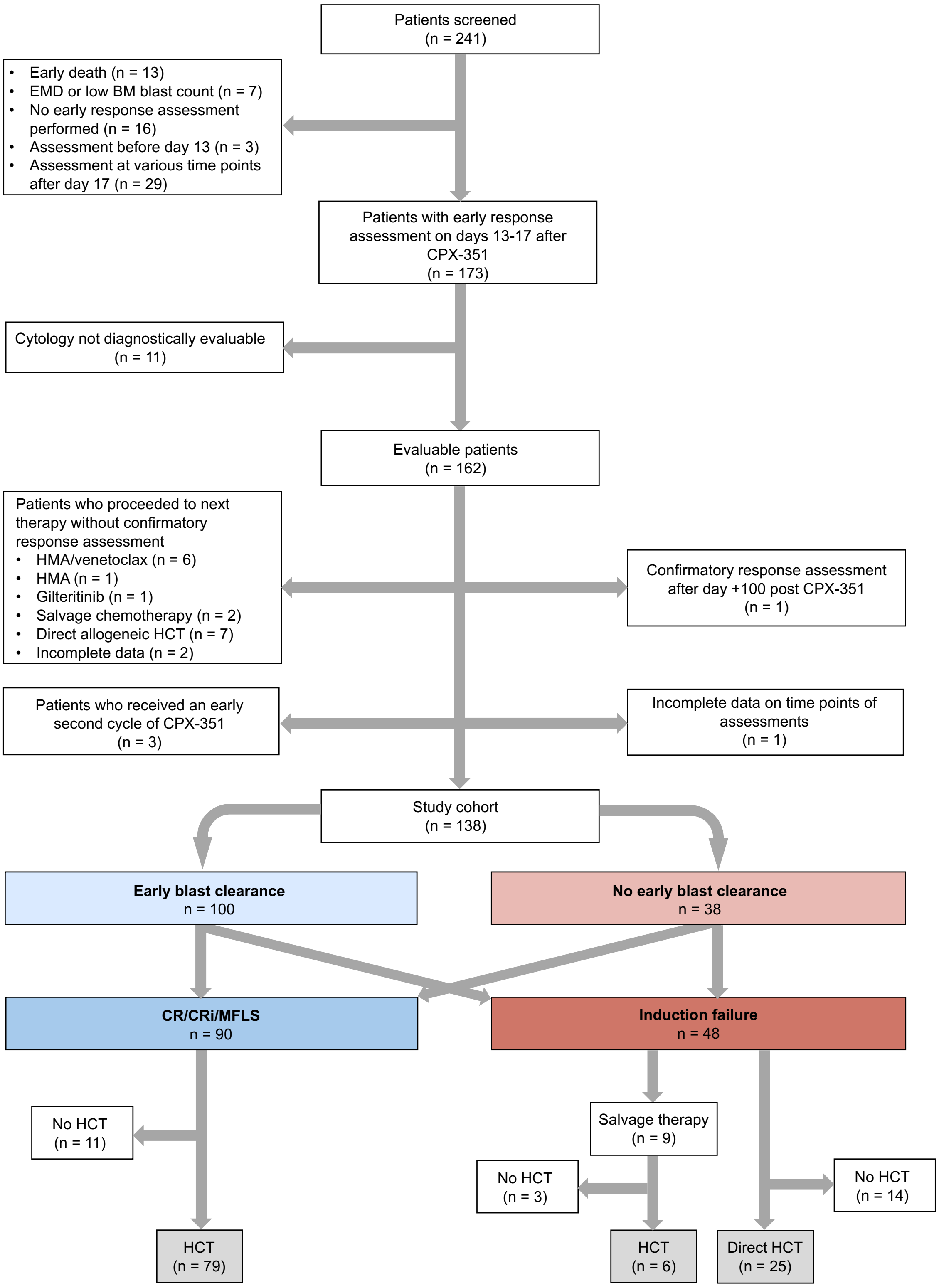


**Supplementary Figure S1 | Patient flow.** Of 173 patients with early response assessment after CPX-351, 138 were finally included in our study. BM, bone marrow. CR, complete remission. CRi, CR with incomplete hematological recovery. EMD, extramedullary disease.

HCT, allogeneic hematopoietic cell transplantation. HMA, hypomethylating agent. MLFS, morphologic leukemia-free state.

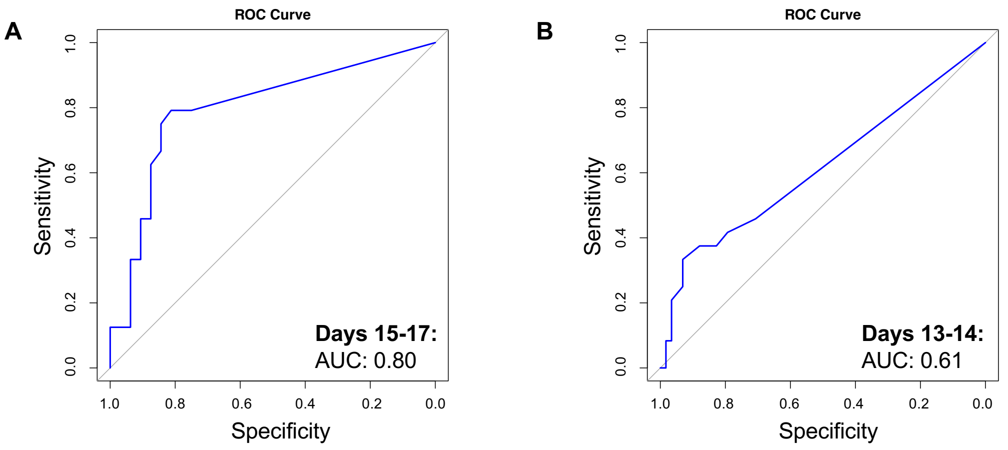


**Supplementary Figure S2 | Impact of timing on the predictive value of early response assessment.** Receiver operating characteristic (ROC) analyses comparing the predictive accuracy of early bone marrow assessments performed on days 15-17 (**A**, n = 57) versus days 13-14 (**B**, n = 84). The area under the curve (AUC) for predicting induction failure (IF) was significantly higher for assessments conducted on days 15–17 (*P* = 0.04, DeLong's test). AUC, area under the curve.

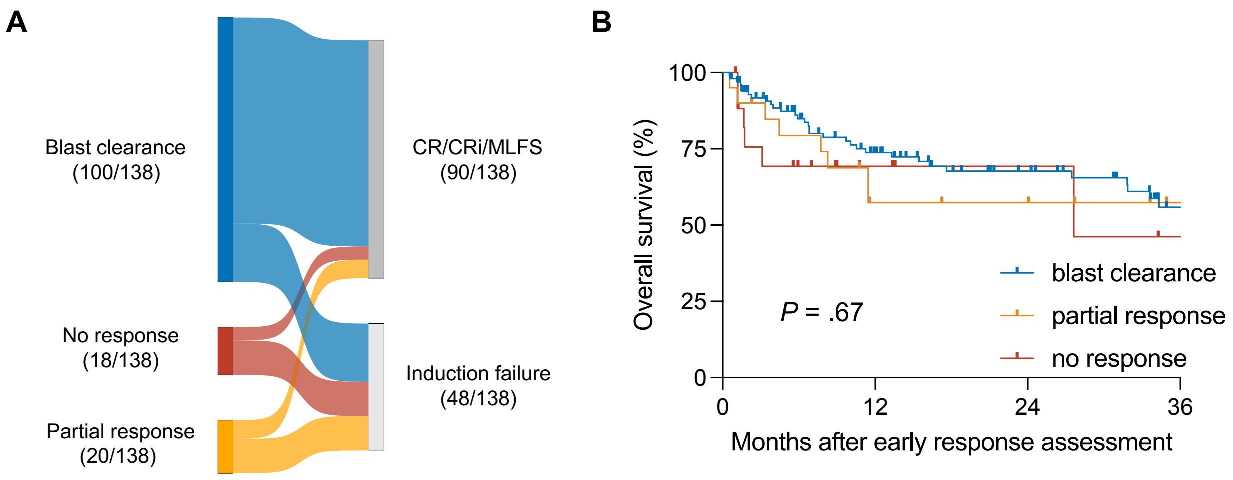


**Supplementary Figure S3 | Outcome of patients with partial response at early response assessment.** Of 20 patients with partial response (according to the definition in Lancet et al. (2), 7 patients achieved either CR, CRi or MLFS after one course of CPX-351 (**A**). OS of patients with partial response did not significantly differ from that of patients with early blast clearance or those without blast clearance or partial response on early response assessment (**B**).

CR, complete remission. CRi, CR with incomplete hematologic recovery. MLFS, morphologic leukemia-free state.

**Supplementary Table 1. Test statistics for the prediction of induction failure based on early response assessment.**

| | **Blast clearance** | **No blast clearance** | |
|---|---|---|---|
| **IF** | 22 | 26 | **Sensitivity** 26/48 (54%) |
| **CR/CRi/MLFS** | 78 | 12 | **Specificity** 78/90 (87%) |
| | **NPV** 78/100 (78%) | **PPV** 26/38 (68%) | |

CR, complete remission. CRi, CR with incomplete haematologic recovery. IF, induction failure. MLFS, morphologic leukaemia-free state. NPV, negative predictive value. PPV, positive predictive value.

**Supplementary Table 2. Test statistics for the prediction of induction failure based on early response assessment, exluding patients with early death (n = 133).**

| | **Blast clearance** | **No blast clearance** | |
|---|---|---|---|
| **IF** | 20 | 23 | **Sensitivity** 23/43 (53%) |
| **CR/CRi/MLFS** | 78 | 12 | **Specificity** 78/90 (87%) |
| | **NPV** 78/98 (80%) | **PPV** 23/35 (66%) | |

CR, complete remission. CRi, CR with incomplete hematological recovery. IF, induction failure. MLFS, morphologic leukemia-free state. NPV, negative predictive value. PPV, positive predictive value.

**Supplementary Table 3. Test statistics for the prediction of induction failure based on early response assessment, using an alternative cut-off of <15% BM blasts to define early blast clearance.**

| | Blast clearance | No blast clearance | |
|---|---|---|---|
| **IF** | 35 | 13 | **Sensitivity** 35/48 (73%) |
| **CR/CRi/MLFS** | 84 | 6 | **Specificity** 84/90 (93%) |
| | **NPV** 84/119 (71%) | **PPV** 13/19 (68%) | |

CR, complete remission. CRi, CR with incomplete hematological recovery. IF, induction failure. MLFS, morphologic leukemia-free state. NPV, negative predictive value. PPV, positive predictive value.

**Supplementary Table 4. Multivariable analysis for induction failure and overall survival.**

| | IF | | | OS | | |
|---|---|---|---|---|---|---|
| | **OR** | **95% CI** | ***P*** | **HR** | **95% CI** | ***P*** |
| **Age**, per 10-year increase | 0.73 | 0.46-1.14 | 0.16 | 1.42 | 0.97-2.07 | 0.07 |
| **Early response assessment** | | | | | | |
| blast clearance | Ref | | | Ref | | |
| no blast clearance | 8.06 | 3.50-19.67 | **< 0.001** | 1.26 | 0.67-2.36 | 0.48 |
| **AML ontogeny** | | | | | | |
| AML-MRC | Ref | | | Ref | | |
| t-AML | 0.58 | 0.20-1.57 | 0.30 | 1.11 | 0.55-2.23 | 0.77 |
| **ELN 2022 genetic risk** | | | | | | |
| non-adverse | Ref | | | Ref | | |
| adverse | 1.09 | 0.41-2.98 | 0.87 | 1.24 | 0.62-2.46 | 0.55 |

AML, acute myeloid leukaemia. AML-MRC, AML with myelodysplasia-related changes. CI, confidence interval. ELN, European LeukemiaNet. HR, hazard ratio. IF, induction failure. OS, overall survival. OR, odds ratio. t-AML, therapy-related AML.

**Supplementary Table 5. Multivariable analysis for induction failure and overall survival, including the BM blast count at early response assessment as a continuous variable.**

| | IF | | | OS | | |
|---|---|---|---|---|---|---|
| | **OR** | **95% CI** | ***P*** | **HR** | **95% CI** | ***P*** |
| **Age**, per 10-year increase | 0.74 | 0.47-1.12 | 0.16 | 1.40 | 0.96-2.05 | 0.08 |
| **Early response assessment,** per 10% increase in BM blasts | 1.65 | 1.23-2.38 | **0.003** | 1.06 | 0.89-1.27 | 0.50 |
| **AML ontogeny** | | | | | | |
| AML-MRC | Ref | | | Ref | | |
| t-AML | 0.69 | 0.26-1.75 | 0.45 | 1.14 | 0.56-2.28 | 0.73 |
| **ELN 2022 genetic risk** | | | | | | |
| non-adverse | Ref | | | Ref | | |
| adverse | 1.39 | 0.56-3.68 | 0.49 | 1.26 | 0.64-2.49 | 0.51 |

AML, acute myeloid leukaemia. AML-MRC, AML with myelodysplasia-related changes. CI, confidence interval. ELN, European LeukemiaNet. HR, hazard ratio. IF, induction failure. OS, overall survival. OR, odds ratio. t-AML, therapy-related AML.

**Supplementary Table 6. Test statistics for the prediction of induction failure based on response assessment conducted on days 15-17 (n = 56).**

| | **Blast clearance** | **No blast clearance** | |
|---|---|---|---|
| **IF** | 7 | 17 | **Sensitivity:** 17/24 (71%) |
| **CR/CRi/MLFS** | 27 | 5 | **Specificity:** 27/32 (84%) |
| | **NPV** 27/34 (79%) | **PPV** 17/22 (77%) | |

CR, complete remission. CRi, CR with incomplete hematological recovery. IF, induction failure. MLFS, morphologic leukemia-free state. NPV, negative predictive value. PPV, positive predictive value.

**Supplementary Table 7. Test statistics for the prediction of induction failure based on response assessment conducted on days 13-14 (n = 82).**

| | **Blast clearance** | **No blast clearance** | |
|---|---|---|---|
| **IF** | 15 | 9 | **Sensitivity:** 9/24 (38%) |
| **CR/CRi/MLFS** | 51 | 7 | **Specificity:** 51/58 (88%) |
| | **NPV** 51/66 (77%) | **PPV** 9/16 (56%) | |

CR, complete remission. CRi, CR with incomplete hematological recovery. IF, induction failure. MLFS, morphologic leukemia-free state. NPV, negative predictive value. PPV, positive predictive value.
